# A guide to selecting high-performing antibodies for S1PR1 (UniProt ID: P21453) for use in western blot, immunoprecipitation, and immunofluorescence

**DOI:** 10.12688/f1000research.153244.3

**Published:** 2025-01-24

**Authors:** Riham Ayoubi, Maryam Fotouhi, Charles Alende, Sara González Bolívar, Kathleen Southern, Carl Laflamme, NeuroSGC/YCharOS/EDDU collaborative group, ABIF consortium

**Affiliations:** 1 Department of Neurology and Neurosurgery, Structural Genomics Consortium, The Montreal Neurological Institute, McGill University, Montreal, Québec, H3A 2B4, Canada

**Keywords:** UniProt ID P21453, S1PR1, sphingosine 1-phosphate receptor 1, EDG1, antibody characterization, antibody validation, western blot, immunoprecipitation, immunofluorescence

## Abstract

Sphingosine 1-phosphate receptor 1 (S1PR1) is a G-coupled protein receptor that induces crucial biological processes when bound by sphingosine 1-phosphate. Here, we have characterized nine S1PR1 commercial antibodies for western blot, immunoprecipitation, and immunofluorescence using a standardized experimental protocol based on comparing read-outs in knockout cell lines and isogenic parental controls. These studies are part of a larger, collaborative initiative seeking to address antibody reproducibility issues by characterizing commercially available antibodies for human proteins and publishing the results openly as a resource for the scientific community. While use of antibodies and protocols vary between laboratories, we encourage readers to use this report as a guide to select the most appropriate antibodies for their specific needs.

## Introduction

Sphingosine 1-phosphate receptor 1 (S1PR1), is a G-protein coupled receptor that binds to its abundant ligand, sphingosine 1-phosphate, inducing intracellular signalling pathways related to cell growth, differentiation, migration and trafficking. ^1^^–^^4^ Also referred to as endothelial differentiation gene 1, S1PR1 plays a role in regulating endothelial cell behaviour, maintaining vascular integrity and promoting angiogenesis. Additionally, it regulates inflammatory responses by modulating T-cell trafficking. ^3^^,^^5^

S1PR1 activation by sphingosine 1-phosphate is essential for neuronal events, and its dysregulation may contribute to the pathogenesis of Alzheimer’s disease. ^6^^,^^7^ A promising target for treating a variety of human diseases, the availability of high-performing S1PR1 antibodies would facilitate S1PR1 research and help uncover therapeutic strategies. ^3^

This research is part of a broader collaborative initiative in which academics, funders and commercial antibody manufacturers are working together to address antibody reproducibility issues by characterizing commercial antibodies for human proteins using standardized protocols, and openly sharing the data. ^8^^–^^10^ Here we evaluated the performance of nine commercial antibodies for S1PR1 for use in western blot, immunoprecipitation, and immunofluorescence, enabling biochemical and cellular assessment of S1PR1 properties and function. The platform for antibody characterization used to carry out this study was endorsed by a committee of industry and academic representatives. It involves identifying appropriate cell lines with adequate target protein expression, developing or contributing equivalent knockout (KO) cell lines and finally, characterizing most commercially available antibodies against the corresponding target protein. The standardized antibody characterization protocols are openly available on Protocol Exchange (DOI: 10.21203/rs.3.pex-2607/v1). ^11^

The authors do not engage in result analysis or offer explicit antibody recommendations. A limitation of this study is the use of universal protocols - any conclusions remain relevant within the confines of the experimental setup and cell line used in this study. Our primary aim is to deliver top-tier data to the scientific community, grounded in Open Science principles. This empowers experts to interpret the characterization data independently, enabling them to make informed choices regarding the most suitable antibodies for their specific experimental needs. Guidelines on how to interpret antibody characterization data found in this study are featured on the YCharOS gateway. ^12^

## Results and discussion

Our standard protocol involves comparing readouts from WT (wild type) and KO cells. ^13^^,^^14^ The first step is to identify a cell line(s) that expresses sufficient levels of S1PR1 to generate a measurable signal using antibodies. To this end, we examined the DepMap transcriptomics database to identify all cell lines that express the target at levels greater than 2.5 log _2_ (transcripts per million “TPM” + 1), which we have found to be a suitable cut-off (Cancer Dependency Map Portal, RRID:SCR_017655). The SK-HEP-1 cells expresses the S1PR1 transcript at 6.5 log _2_ (TPM+1) RNA levels, which is above the average range of cancer cells analyzed. The parental SK-HEP-1 cell line was obtained from the American Type Culture Collection (ATCC) while *S1PR1* KO SK-HEP-1 cells, prepared using CRISPR/Cas9 technology, was custom made from Abcam ( Table 1).

**Table 1.** Summary of the cell lines used.

Institution	Catalog number	RRID (Cellosaurus)	Cell line	Genotype
ATCC	HTB-52	CVCL_0525	SK-HEP-1	WT
Abcam	-	-	SK-HEP-1	*S1PR1* KO

**Figure 1.**
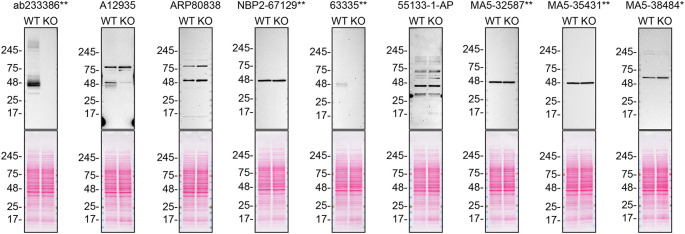
S1PR1 antibody screening by western blot. Lysates of SK-HEP-1 (WT and *S1PR1* KO) were prepared and 30 μg of protein were processed for western blot with the indicated S1PR1 antibodies. The Ponceau stained transfers of each blot are presented to show equal loading of WT and KO lysates and protein transfer efficiency from precast midi 4-20% Tris-Glycine polyacrylamide gel (Thermo Fisher Scientific, cat number WXP42012BOX) to the nitrocellulose membrane. Antibody dilutions were chosen according to the recommendations of the antibody supplier. Antibody dilution used: ab233386** at 1/1000, A12935 at 1/1000, ARP80838 at 1/500., NBP2-67129** at 1/1000, 63335** at 1/1000, 55133-1-AP at 1/1000, MA5-32587** at 1/1000, MA5-35431** at 1/1000, MA5-38484* at 1/500. Predicted band size: 42.8 kDa. *Monoclonal antibody, **Recombinant antibody. A repeat experiment is available as Extended data – Figure 1.

For western blot experiments, WT and *S1PR1* KO protein lysates were rain on SDS-PAGE, transferred onto nitrocellulose membranes, and then probed with nine S1PR1 antibodies ( Table 2) in parallel ( Figure 1).

**Table 2.** Summary of the S1PR1 antibodies tested.

Company	Catalog number	Lot number	RRID (Antibody Registry)	Clonality	Clone ID	Host	Concentration (μg/μl)	Vendors recommended applications
Abcam	ab233386 **	GR3404607-2	AB_2928162	recombinant-mono	EPR21202	rabbit	0.43	Wb
ABclonal	A12935	59370101	AB_2759781	polyclonal	-	rabbit	1.10	Wb
Aviva Systems Biology	ARP80838	QC56393-190325	AB_3083071	polyclonal	-	rabbit	0.50	Wb
Novus Biologicals (a Bio-Techne brand)	NBP2-67129 **	HN1019	AB_3083072	recombinant-mono	JM10-66	rabbit	1.00	Wb, IF
Cell Signaling Technology	63335 **	1	AB_3083073	recombinant-mono	E8U3O	rabbit	0.20	Wb, IP, IF
Proteintech	55133-1-AP	89828	AB_10793721	polyclonal	-	rabbit	1.00	Wb, IP
Thermo Fisher Scientific	MA5-32587 **	XC3523726	AB_2809864	recombinant-mono	JM10-66	rabbit	1.00	Wb, IF
Thermo Fisher Scientific	MA5-35431 **	XC3523881	AB_2849332	recombinant-mono	ARC0881	rabbit	0.29	Wb
Thermo Fisher Scientific	MA5-38484 *	XC3523353	AB_2898397	monoclonal	8EAH5	mouse	1.00	Wb

Wb = western blot; IF = immunofluorescence; IP = immunoprecipitation.

* Monoclonal antibody.

** Recombinant antibody.

**Figure 2.**
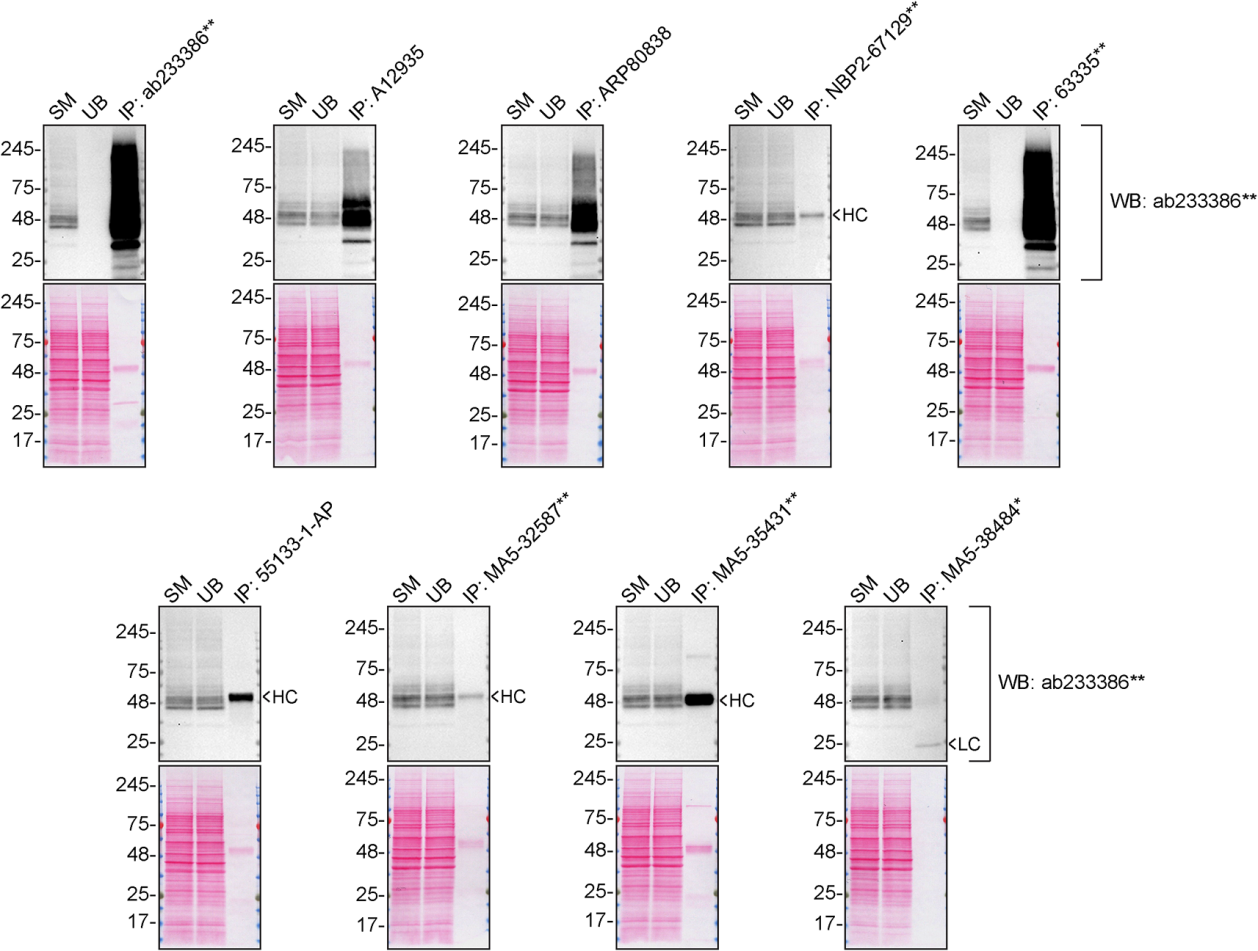
S1PR1 antibody screening by immunoprecipitation. SK-HEP-1 lysates were prepared, and immunoprecipitation was performed using 2.0 μg of the indicated S1PR1 antibodies pre-coupled to Dynabeads protein A or protein G. Samples were washed and processed for western blot on a precast midi 4-20% Tris-Glycine polyacrylamide gel with the indicated S1PR1 antibodies. For western blot, ab233386** was used at 1/1000. The Ponceau stained transfers of each blot are shown. SM = 4% starting material; UB = 4% unbound fraction; IP = immunoprecipitate, HC = antibody heavy chain, LC = antibody light chain. *Monoclonal antibody, **Recombinant antibody.

We then assessed the capability of all nine antibodies to capture S1PR1 from SK-HEP-1 protein extracts using immunoprecipitation techniques, followed by western blot analysis. For the immunoblot step, a specific S1PR1 antibody identified previously ( Figure 1) was selected. Equal amounts of the starting material (SM), the unbound fraction (UB), as well as the whole immunoprecipitate (IP) eluates were separated by SDS-PAGE ( Figure 2).

**Figure 3.**
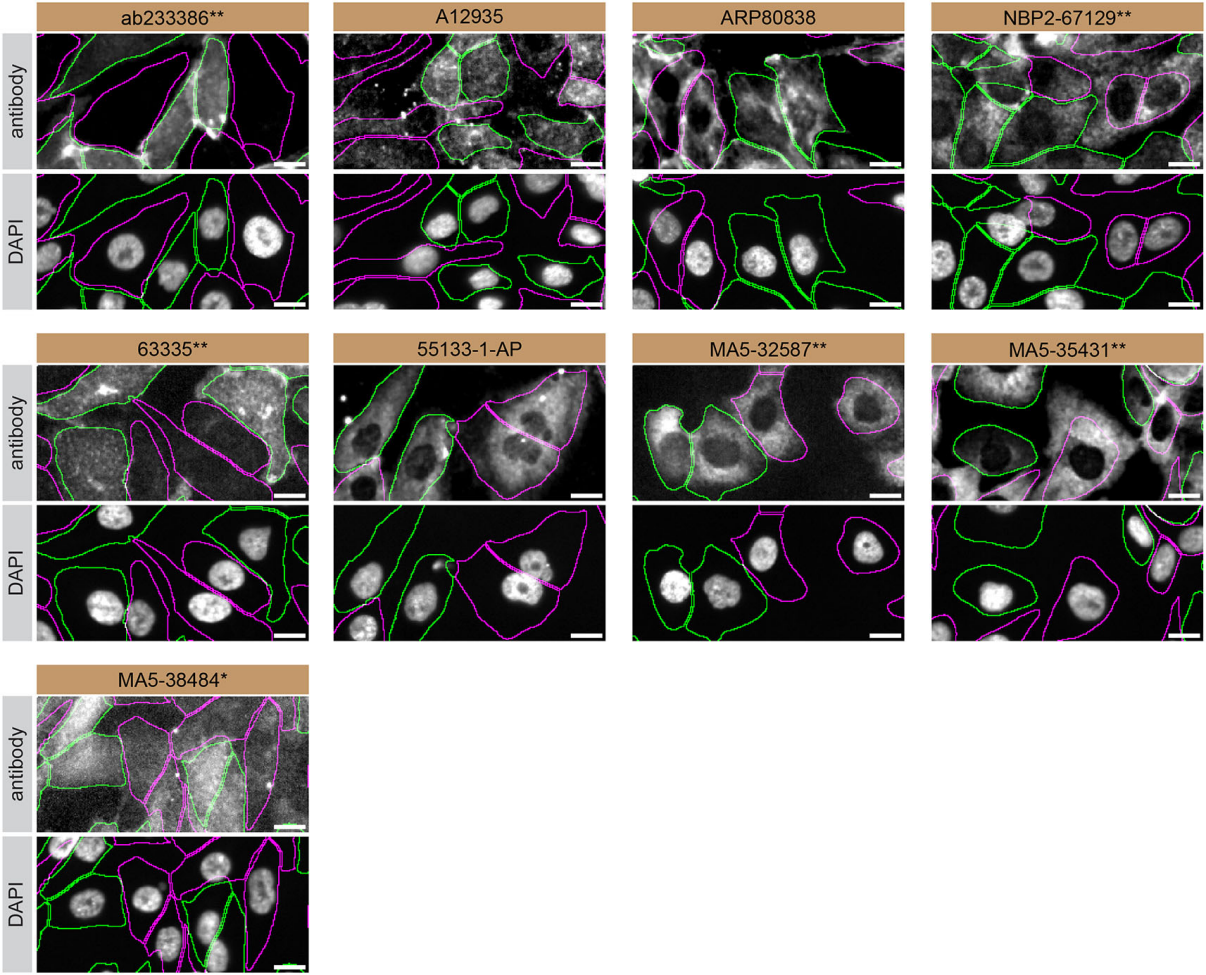
S1PR1 antibody screening by immunofluorescence. SK-HEP-1 WT and *S1PR1* KO cells were labelled with a green or a far-red fluorescent dye, respectively. WT and KO cells were mixed and plated to a 1:1 ratio in a 96-well plate with optically clear flat-bottom. Cells were stained with the indicated S1PR1 antibodies and with the corresponding Alexa-fluor 555 coupled secondary antibody including DAPI. Acquisition of the blue (nucleus-DAPI), green (identification of WT wells), red (antibody staining) and far-red (identification of KO cells) channels was performed. Representative images of the merged blue and red (grayscale) channels are shown. WT and KO cells are outlined with green and magenta dashed line, respectively. When an antibody was recommended for immunofluorescence by the supplier, we tested it at the recommended dilution. The rest of the antibodies were tested at 1 and 2 μg/ml and the final concentration was selected based on the detection range of the microscope used and a quantitative analysis not shown here. Antibody dilution used: ab233386** at 1/400, A12935 at 1/1000, ARP80838 at 1/250., NBP2-67129** at 1/1000, 63335** at 1/2000, 55133-1-AP at 1/500, MA5-32587** at 1/1000, MA5-35431** at 1/300, MA5-38484* at 1/200. Bars = 10 μm. *Monoclonal antibody, **Recombinant antibody. Antibody performance is available in Extended data – Figure 2.

For immunofluorescence, nine antibodies were screened using a mosaic strategy. This strategy takes advantage of the WT and KO cells being plated together as a mosaic, to enable imaging within a single field of view. To create this mosaic, the SK-HEP-1 WT and *S1PR1* KO cells were first labelled with different fluorescent dyes in order to distinguish the two cell lines, and the S1PR1 antibodies were evaluated. Both WT and KO cells were imaged in the same field of view to reduce staining, imaging and image analysis bias ( Figure 3). Quantification of immunofluorescence intensity in hundreds of WT and KO cells was performed for each antibody tested, ^11^ and the images presented in Figure 3 are representative of this analysis.

In conclusion, we have screened nine S1PR1 commercial antibodies by western blot, immunoprecipitation, and immunofluorescence by comparing the signal produced by the antibodies in human SK-HEP-1 WT and *S1PR1* KO cells. To guide viewers in assessing the results in each application, Table 3 illustrates the various scenarios in which an antibody can perform and the corresponding outcomes interpreted across all three applications ( Table 3). ^8^ Several high-quality antibodies that successfully detect S1PR1 under our standardized experimental protocol can be identified. Researchers who wish to study S1PR1 in a different species are encouraged to select high-quality antibodies, based on the results of this study, and investigate the predicted species reactivity of the manufacturer before extending their research.

**Table 3.** Illustrations to assess antibody performance in all three applications.

Western blot	Immunoprecipitation	Immunofluorescence
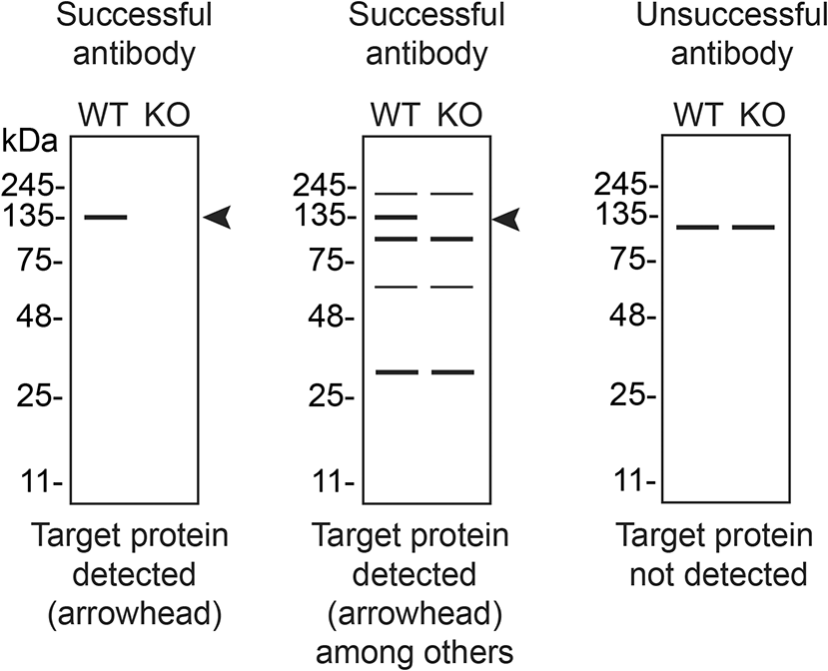	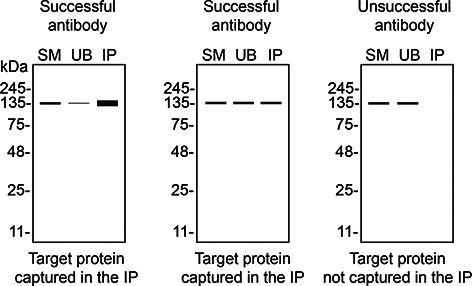	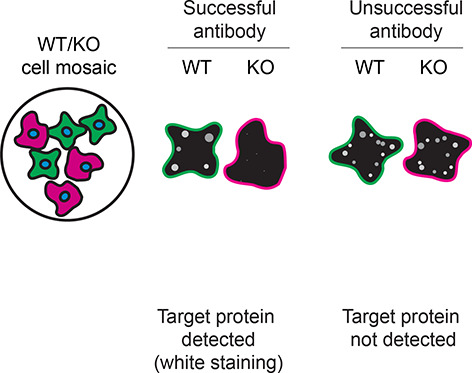

This table has been reproduced with permission from Ayoubi et al., Elife, 2023 ^8^ (CC BY lisence).

The underlying data for this study can be found on Zenodo, an open-access repository for which YCharOS has its own collection of antibody characterization reports. ^18^^,^^19^

## Methods

The standardized protocols used to carry out this KO cell line-based antibody characterization platform was established and approved by a collaborative group of academics, industry researchers and antibody manufacturers. The detailed materials and step-by-step protocols used to characterize antibodies in western blot, immunoprecipitation and immunofluorescence are openly available on Protocol Exchange, a preprint server (DOI: 10.21203/rs.3.pex-2607/v1). ^11^ Brief descriptions of the experimental setup used to carry out this study can be found below.

### Antibodies and cell line used

Cell lines used and primary antibodies tested in this study are listed in Tables 1 and 2, respectively. To ensure that the cell lines and antibodies are cited properly and can be easily identified, we have included their corresponding Research Resource Identifiers, or RRID. ^15^^,^^16^ SK-HEP-1 KO clone corresponding to the *S1PR1* gene was generated with low passage cells at Abcam. Two guide RNAs were used to induce a deletion in the *S1PR1* gene (sequence guide 1: TCAACTATGATATCATCGTC, sequence guide 2: GCTGAATATCAGCGCGGACA). Peroxidase-conjugated goat anti-rabbit and anti-mouse are from Thermo Fisher Scientific (cat. number 65-6120 and 62-6520). Protein A:HRP is from MilliporeSigma (cat. number P8651).

### Antibody screening by western blot

SK-HEP-1 WT and *S1PR1* KO (listed in Table 1) were collected in RIPA buffer (25mM Tris-HCl pH 7.6, 150mM NaCl, 1% NP-40, 1% sodium deoxycholate, 0.1% SDS) from Thermo Fisher Scientific (cat. number 89901) supplemented with 1x protease inhibitor cocktail mix (MilliporeSigma, cat. number P8340). Lysates were sonicated briefly, rocked for 30 min in a cold room, then spun at ~110,000 × *g* for 15 min at 4°C. Equal aliquots of 30 μg supernatants were analyzed by SDS-PAGE and western blot. BLUelf prestained protein ladder from GeneDireX (cat. number PM008-0500) was used.

Western blots were performed with precast midi 4-20% Tris-Glycine polyacrylamide gels from Thermo Fisher Scientific (cat. number WXP42012BOX) ran with Tris/Glycine/SDS buffer from Bio-Rad (cat. number 1610772), loaded in Laemmli loading sample buffer from Thermo Fisher Scientific (cat. number AAJ61337AD) and transferred on nitrocellulose membranes. Blots were blocked with 5% milk for 1 hr, and antibodies were incubated O/N at 4°C with 5% milk in TBS with 0.1% Tween 20 (TBST) from Cell Signaling Technology (cat. number 9997). Following three washes with TBST, the peroxidase conjugated secondary antibody was incubated at a dilution of ~0.2 μg/ml in TBST with 5% milk for 1 hr at room temperature followed by three washes with TBST. Membranes were incubated with Pierce ECL from Thermo Fisher Scientific (cat. number 32106) or Clarity Western ECL Substrate from Bio-Rad (cat. number 1705061) prior to detection with the iBright™ CL1500 Imaging System from Thermo Fisher Scientific (cat. number A44240).

### Antibody screening by immunoprecipitation

Antibody-beads conjugates were prepared by adding 2.0 μg to 500 μl of Pierce IP Lysis Buffer from Thermo Fisher Scientific (cat. number 87788) in a microcentrifuge tube, together with 30 μl of Dynabeads protein A- (for rabbit antibodies) or protein G- (for mouse antibodies) from Thermo Fisher Scientific (cat. number 10002D and 10004D, respectively). Tubes were rocked for ~1 hr at 4°C followed by two washes to remove unbound antibodies.

SK-HEP-1 WT were collected in Pierce IP buffer (25 mM Tris-HCl pH 7.4, 150 mM NaCl, 1 mM EDTA, 1% NP-40 and 5% glycerol) supplemented with protease inhibitor. Lysates were rocked for 30 min at 4°C and spun at 110,000 × *g* for 15 min at 4°C. 0.5 ml aliquots at 2.0 mg/ml of lysate (1 mg) were incubated with an antibody-bead conjugate for ~1 hr at 4°C. The unbound fractions were collected, and beads were subsequently washed three times with 1.0 ml of IP lysis buffer and processed for SDS-PAGE and western blot on precast midi 4-20% Tris-Glycine polyacrylamide gels. Protein A:HRP (MilliporeSigma, cat. number P8651) was used as a secondary detection system at a concentration of 2.0 μg/ml.

### Antibody screening by immunofluorescence

SK-HEP-1 WT and *S1PR1* KO were labelled with a green and a far-red fluorescence dye, respectively. The fluorescent dyes used are from Thermo Fisher Scientific (cat. number C2925 and C34565). WT and KO cells were plated in a 96-well plate with optically clear flat-bottom (Perkin Elmer, cat. number 6055300) as a mosaic and incubated for 24 hrs in a cell culture incubator. Cells were fixed in 4% PFA (in PBS) for 15 min at room temperature and then washed 3 times with PBS. Cells were permeabilized in PBS with 0,1% Triton X-100 for 10 min at room temperature and blocked with PBS with 5% BSA, 5% goat serum and 0.01% Triton X-100 for 30 min at room temperature. Cells were incubated with IF buffer (PBS, 5% BSA, 0,01% Triton X-100) containing the primary S1PR1 antibodies overnight at 4°C. Cells were then washed 3 × 10 min with IF buffer and incubated with corresponding Alexa Fluor 555-conjugated secondary antibodies in IF buffer at a dilution of 1.0 μg/ml for 1 hr at room temperature with DAPI. Cells were washed 3 × 10 min with IF buffer and once with PBS.

Images were acquired on an ImageXpress micro confocal high-content microscopy system (Molecular Devices), using a 20× NA 0.95 water immersion objective and scientific CMOS cameras, equipped with 395, 475, 555 and 635 nm solid state LED lights (lumencor Aura III light engine) and bandpass filters to excite DAPI, Cellmask Green, Alexa-555 and Cellmask Red, respectively. Images had pixel sizes of 0.68 × 0.68 microns, and a z-interval of 4 microns. For analysis and visualization, shading correction (shade only) was carried out for all images. Then, maximum intensity projections were generated using 3 z-slices. Segmentation was carried out separately on maximum intensity projections of Cellmask channels using CellPose 1.0, and masks were used to generate outlines and for intensity quantification. Figures were assembled with Adobe Illustrator. ^17^

### Limitations

Inherent limitations are associated with the antibody characterization platform employed in this study. ^11^ The authors do not claim to have expertise in S1PR1 or sphingosine-1-phosphate signalling mechanisms, which is why a brief background of the protein’s function and relevance in disease is provided. Adopting an agnostic approach, the authors perform antibody-based applications and share the results openly, leaving the analysis and interpretation up to the readers.

For the YCharOS effort, experiments are not commonly performed in replicates. The rationale behind this approach is related to the fact that the validation of the KO cell lines involves the use of multiple antibodies targeting various epitopes. Once a specific antibody is identified, it validates the protein expression of the intended target in the selected cell line at a concentration that is detectible by a suitable antibody and supports conclusions regarding the specificity of the other antibodies. Typically, we have access to the same antibody from 2-3 manufacturers (cross-licensed antibodies), which effectively serve as replicates, enabling the validation of test reproducibility. All experiments are performed using master mixes, and meticulous attention is paid to sample preparation and experimental execution. In IF, the use of two different concentrations serves to evaluate antibody specificity and can aid in assessing assay reliability. In instances where antibodies yield no signal, a repeat experiment is conducted. A repeat of the western blot screening experiment, along with the analysis of antibody performance in immunofluorescence across thousands of cells, using the analysis code available on GitHub, is available as extended data on Zenodo.

As comprehensive and standardized procedures are respected, any conclusions remain confined to the experimental conditions and cell line used for this study. The use of a single cell line for evaluating antibody performance poses as a limitation, as factors such as target protein abundance significantly impact results. Additionally, the use of cancer cell lines containing gene mutations poses a potential challenge, as these mutations may be within the epitope coding sequence or other regions of the gene responsible for the intended target. Such alterations should impact the binding affinity of antibodies. This represents an inherent limitation of any approach that employs cancer cell lines.

## Data Availability

Zenodo: Antibody Characterization Report for S1PR1, doi.org/10.5281/zenodo.10819189. ^18^ Zenodo: Dataset for the S1PR1 antibody screening study, doi.org/10.5281/zenodo.10839647. ^19^ Data are available under the terms of the Creative Commons Attribution 4.0 International license (CC-BY 4.0). A repeat experiment of the S1PR1 antibody screening in western blot, as well as the analysis of the analysis of antibody performance in immunofluorescence across thousands of cells can be found on Zenodo: https://doi.org/10.5281/zenodo.14447748. The imaging analysis codes are available on GitHub: https://github.com/ABIF-McGill/YCharOS_IF_characterization/.
