# Supplementary material for: A guide to selecting high-performing antibodies for S1PR1 (UniProt ID: P21453) for use in western blot, immunoprecipitation, and immunofluorescence
Source: F1000Res. 2025 Jan 24;13:792. Originally published 2024 Jul 11. [Version 3] doi: 10.12688/f1000research.153244.3 (PMC12022542; doi:10.12688/f1000research.153244.3)
Supplement: Peer review history

# Reviewer report: Reviewer response for version 3

**DOI:** 10.5256/f1000research.175985.r371792

**Published:** 2025-04-24

**Authors:** Jieya Shao

**Affiliations:** 1 Washington University in St Louis, St. Louis, USA

In this article the authors systematically compared the performance and specificity of nine commercial anti-S1PR1 antibodies using three standard experimental approaches (Western blot, immunoprecipitation, immunofluorescence staining). This study is part of a large collaborative initiative to characterize commercial antibodies for different human proteins and offer unbiased guidance to researchers who can select the best antibodies for their specific experimental objectives based on their own interpretations. As a Data Note, this paper successfully achieved its goal with rigorously collected high-quality data, and the results are self-explanatory and useful for the research community.

 One typo was found on page 3 in the first sentence of the last paragraph. The word "rain" seems to be mistakenly used instead of "run". Another minor suggestion regarding Figure 3 is that the authors can arrange the nine panels into a 3x3 format which makes full use of the space.

Are sufficient details of methods and materials provided to allow replication by others?

Yes

Is the rationale for creating the dataset(s) clearly described?

Yes

Are the datasets clearly presented in a useable and accessible format?

Yes

Are the protocols appropriate and is the work technically sound?

Yes

Reviewer Expertise:

DNA damage response, DNA replication stress, cancer biology, chemotherapy

I confirm that I have read this submission and believe that I have an appropriate level of expertise to confirm that it is of an acceptable scientific standard.

# Reviewer report: Reviewer response for version 2

**DOI:** 10.5256/f1000research.170465.r328885

**Published:** 2024-11-04

**Authors:** Christian Tiede

**Affiliations:** 1 University of Leeds, Leeds, England, UK

In this article, nine anti-S1PR1 antibodies are evaluated in WB, IP, and IF using wt and knockout SK-HEP-1 cells. The article provides a useful resource for other scientists in selecting antibodies for their applications. The methods are detailed and well described and easy for the reader to repeat. It is indeed disappointing, as already described by the previous reviewer, that in the IP the secondary detection reagents (ProteinA-HRP, MilliporeSigma, cat. number P8651) not only detect the primary antibody (Abcam, ab233386), but also the denatured heavy and light chain of the IP antibody. For future experiments, direct immobilization of the IP antibodies on the beads via free amines and elution at pH2 could be considered. However, in this study, despite being similar in size to the S1PR1 protein, it does not appear that detecting the heavy chain would change the conclusion of the experiment. I therefore recommend approval.

Are sufficient details of methods and materials provided to allow replication by others?

Yes

Is the rationale for creating the dataset(s) clearly described?

Yes

Are the datasets clearly presented in a useable and accessible format?

Yes

Are the protocols appropriate and is the work technically sound?

Yes

Reviewer Expertise:

Phage display

I confirm that I have read this submission and believe that I have an appropriate level of expertise to confirm that it is of an acceptable scientific standard.

# Reviewer report: Reviewer response for version 2

**DOI:** 10.5256/f1000research.170465.r321079

**Published:** 2024-09-19

**Authors:** Melissa Pitman

**Affiliations:** 1 University of Adelaide, The University of Adelaide Faculty of Sciences (Ringgold ID: 98478), Adelaide, South Australia, Australia

The authors have tried to address all of the comments. They have clearly stated the limitations to their study.

 For the record, while I agree that conducting orthologous experiments and being careful with experimental set-ups can help reduce variability, I would argue that biological experiments will nearly always have some variability and should always be repeated more than once.

Are sufficient details of methods and materials provided to allow replication by others?

No

Is the rationale for creating the dataset(s) clearly described?

Yes

Are the datasets clearly presented in a useable and accessible format?

Partly

Are the protocols appropriate and is the work technically sound?

Partly

Reviewer Expertise:

I work across cancer cell biology, biochemistry and molecular biology and have experience with western blotting, immunoprecipitation and immunofluorescence techniques.

I confirm that I have read this submission and believe that I have an appropriate level of expertise to confirm that it is of an acceptable scientific standard, however I have significant reservations, as outlined above.

# Response

**Published:** 2024-12-13

**Authors:** Carl Laflamme

**Affiliations:** McGill University, Canada

Dear Dr. Pitman,

 Thank you for your follow-up comments regarding experimental reproducibility. We have repeated the antibody screening and, as detailed in the extended data available on Zenodo (with the link provided in the revised manuscript), we include the full antibody screening results in western blot and an analysis of antibody performance in immunofluorescence, conducted on at least 500 wild-type (WT) and knockout (KO) cells per condition.

 With improved titration, antibody 63335 now demonstrates a higher signal-to-noise ratio than previously shown and is fully suitable for detecting S1PR1 in western blot. The immunofluorescence analysis provides a more direct comparison of performance between antibodies and highlights the variability of S1PR1 expression across the cell population.

 We hope these additional data adequately address your concerns.

 Best regards,

 Carl Laflamme

# Reviewer report: Reviewer response for version 1

**DOI:** 10.5256/f1000research.168111.r302715

**Published:** 2024-08-05

**Authors:** Melissa Pitman

**Affiliations:** 1 University of Adelaide, The University of Adelaide Faculty of Sciences (Ringgold ID: 98478), Adelaide, South Australia, Australia

This paper used the WT and S1PR1 knockout SK-HEP-1 cells to evaluate the quality of the anti-S1PR1 antibodies obtained from different companies in three different applications, i.e. WB, IP, and IF. The results could be a useful guide for other scientists to help identify antibodies that are validated for their experimental application.

 General comments:

 -REQUIRED CHANGE: In the introduction there is one single line that indicates a role for S1PR1 only in neuronal events. S1PR1 has many roles throughout the body and this background needs to reflect that.

 -REQUIRED CHANGE: It is unclear from the methods (in the paper or the linked protocols) and figure legends how many times these experiments were conducted. Please include the number of experiments (n=?) in the figure legends. Three independent experiments is the standard for most scientific studies.

 -There are a number of published papers from the ABIF consortium with the same format. While understandable for such a multi-pronged study, some of the wording is identical between each paper and some of the same issues are present in those other studies. 

 -Although the reason is explicitly stated in the manuscript, it is highly unusual for a study to not explain any interpretations of their data or make any conclusions. 

 -REQUIRED CHANGE: The catalog number for the Abcam S1PR1 KO cells is not included - were these made custom? Please include details on how these were made e.g. CRISPR/Cas9.

 REQUIRED CHANGEs: Specific comments:

 -Results: "nine antibodies were screened using a mosaic strategy." Please add further detail to this sentence as not all readers will know what this strategy means.

 -Figure 1: far right gel. Please check the molecular weight markers are correct on the gel. The numbers appear to be shifted down compared to the other gel images.

 -Figure 2: The immunoprecipitation experiments appear to use an anti-rabbit (ab233386) antibody to detect the S1PR1 in the western blots (after immunoprecipitation). Unfortunately, using anti-rabbit antibodies in both the blots and IPs means that the heavy and light chain will be detected by the secondary antibodies. For S1PR1 that runs around the same size as the heavy chain this means that conclusive information cannot be gained. As stated in your protocol (section 2E), if you do not have an appropriate secondary antibody from a different species then other methods may need to be considered. The authors should consider using an appropriate TrueBlot (Rockland) secondary antibody to avoid detection of heavy chain or test whether running their gels under non-denaturing conditions will move the antibody band out of the same size range as the S1PR1 target.

 -Figure 2 - please include the protein quantity of lysates (ug) for the IP.

Are sufficient details of methods and materials provided to allow replication by others?

No

Is the rationale for creating the dataset(s) clearly described?

Yes

Are the datasets clearly presented in a useable and accessible format?

Partly

Are the protocols appropriate and is the work technically sound?

Partly

Reviewer Expertise:

I work cross cancer cell biology, biochemistry and molecular biology and have experience with western blotting, immunoprecipitation and immunofluorescence techniques.

I confirm that I have read this submission and believe that I have an appropriate level of expertise to state that I do not consider it to be of an acceptable scientific standard, for reasons outlined above.

# Response

**Published:** 2024-08-13

**Authors:** Kathleen Southern

**Affiliations:** McGill University, Canada

Thank you to Melissa Pitman for your comprehensive review of this manuscript. The authors have taken great consideration from all suggested changes and made the necessary changes to ensure future reproducibility of the antibody characterization procedure. That being said, we have replied to all of your comments ( *italicized*) below and created a new version of the manuscript to address all concerns, when appropriate. We hope that the adjustments made meet your expectations and clarify any concerns you previously had. Please see below.

*General comments:*

*-REQUIRED CHANGE: In the introduction there is one single line that indicates a role for S1PR1 only in neuronal events. S1PR1 has many roles throughout the body and this background needs to reflect that.*

 To address this concern, the authors have revisited the introduction to provide further background on the function of S1PR1 throughout the body. Please refer to version 2.

*-REQUIRED CHANGE: It is unclear from the (1)methods (in the paper or the linked protocols) and figure legends how many times these experiments were conducted. Please include the number of experiments (n=?) in the figure legends. Three independent experiments is the standard for most scientific studies.*

 We understand the perceived concern of the lack of replicates in our study. To address this concern, as well as other concerns commonly reported from reviewers, the authors have included a limitation section that will proceed the methods section. To reiterate the rationale behind this selected approach, the validation of the KO cell lines involves the use of multiple antibodies targeting various epitopes. Once a specific antibody is identified, it validates the protein expression of the intended target in the selected cell line at a concentration that is detectible by a suitable antibody and supports conclusions regarding the specificity of the other antibodies. Typically, we have access to the same antibody from 2-3 manufacturers (cross-licensed antibodies), which effectively serve as replicates, enabling the validation of test reproducibility. All experiments are performed using master mixes, and meticulous attention is paid to sample preparation and experimental execution. In IF, the use of two different concentrations serves to evaluate antibody specificity and can aid in assessing assay reliability. In instances where antibodies yield no signal, a repeat experiment is conducted. After careful consideration, it was determined that conducting experiments in replicates does not offer significant benefits in this context. 

*-There are a number of published papers from the ABIF consortium with the same format. While understandable for such a multi-pronged study, some of the wording is identical between each paper and some of the same issues are present in those other studies.*

**

 The YCharOS initiative is a consensus platform developed through collaboration with academic and industry leaders to deliver antibody characterization data for human proteins to the scientific community. The collection of antibody characterization results are seen as a collection for public good, grounded on open science principles, to address research reproducibility issues and help researchers select high-quality antibodies for their experimental needs. That being said, a common template is used to deliver such antibody characterization Data Notes to the community to ensure the procedure followed is consistent and reproducible.

*-Although the reason is explicitly stated in the manuscript, it is highly unusual for a study to not explain any interpretations of their data or make any conclusions.* 

 We understand this concern and it is why we have chosen to submit the antibody characterization data in the format of Data Notes. As explained in the F1000 article guidelines, Data Notes do not include result analyses or conclusions. Furthermore, the authors prefer to remain unbiased while being transparent with the results obtained from the experiments. To provide context for readers who do not have experience analyzing results from such characterization methods utilizing knockout or knockdown lines as controls, we have included a subsequent Table to demonstrate how one can identify successful vs unsuccessful antibodies in each application. Please refer to Table 3 in the updated submission.

*-REQUIRED CHANGE: The catalog number for the Abcam S1PR1 KO cells is not included - were these made custom? Please include details on how these were made e.g. CRISPR/Cas9.*

 The information regarding the SK-HEP-1 *S1PR1* KO cell line provided by Abcam has been included in the Results section. Specific guide RNA sequences can be found in the methods section under “Antibodies and cell line used”. Please refer to version 2.

*REQUIRED CHANGEs: Specific comments:*

*-Results: "nine antibodies were screened using a mosaic strategy." Please add further detail to this sentence as not all readers will know what this strategy means.*

 That you for bringing up this concern. We understand that some readers may not have experience with IF or this method in particular, which is why we have elaborated on what the mosaic strategy entails in the proceeding sentences of the IF paragraph within the results section. The clarification is also provided in the IF methods section. Please refer to version 2.

*-Figure 1: far right gel. Please check the molecular weight markers are correct on the gel. The numbers appear to be shifted down compared to the other gel images.*

 After checking the raw data for antibody MA5-38484* evaluation by western blot (Wb), we can confirm that the placement of the molecular weight markers is correct. This can also be detected by the distinct 75 kDa signal on the left side of the blot. We predict that the proteins did not migrate as far down as intended during the protein separation by SDS-PAGE, which is why the S1PR1 protein was picked up by the antibody at a higher molecular weight. That being said, it is evident from the results that this antibody would not be appropriate to select for Wb analysis (refer to Table 3) given that the signal is not lost in the KO cell line, indicating that the antibody is not specific.

*-Figure 2: The immunoprecipitation experiments appear to use an anti-rabbit (ab233386) antibody to detect the S1PR1 in the western blots (after immunoprecipitation). Unfortunately, using anti-rabbit antibodies in both the blots and IPs means that the heavy and light chain will be detected by the secondary antibodies. For S1PR1 that runs around the same size as the heavy chain this means that conclusive information cannot be gained. As stated in your protocol (section 2E), if you do not have an appropriate secondary antibody from a different species then other methods may need to be considered. The authors should consider using an appropriate TrueBlot (Rockland) secondary antibody to avoid detection of heavy chain or test whether running their gels under non-denaturing conditions will move the antibody band out of the same size range as the S1PR1 target.*

 As stated in the troubleshooting section of the protocol 2E, to avoid cross-reactivity of the antibody with the chains, a secondary detection system that prevents these cross-reactions with unstructured immunoglobulins should be used ( https://doi.org/10.21203/rs.3.pex-2607/v1). That being said, Prot A:HRP secondary detection system was used in this study. This relevant information has been included in the antibody and Wb methods sections. Please refer to version 2.

*-Figure 2 - please include the protein quantity of lysates (ug) for the IP.*

 This information has been added IP methods section. Please refer to version 2.
